# Influence of torque and bone type on stability quotient of two implant platforms: a clinical trial

**DOI:** 10.1590/1807-3107bor-2024.vol38.0049

**Published:** 2024-06-24

**Authors:** Lisiane Cristina BANNWART, Daniela Micheline dos SANTOS, João Paulo do Vale SOUZA, Clóvis Lamartine de Moraes MELO NETO, Emily Vivianne Freitas da SILVA, José Vitor Quinelli MAZARO, Leda Maria Piscinini SALZEDAS, Marcelo Coelho GOIATO

**Affiliations:** (a) Universidade Estadual Paulista – Unesp, Aracatuba Dental School, Department of Dental Materials and Prosthodontics, Aracatuba, SP, Brazil.; (b) Universidade de São Paulo – USP, School of Dentistry, Department of Prosthodontics, São Paulo, SP, Brazil.; (c) Universidade Estadual Paulista – Unesp, Aracatuba Dental School, Department of Diagnosis and Surgery, Aracatuba, SP, Brazil.

**Keywords:** Dental Implants, Cortical Bone, Torque, Alveolar Bone Loss

## Abstract

The objective of this study was to analyze the influence of insertion torque, bone type, and peri-implant bone loss on implant stability quotient (ISQ) of cylindrical external hexagon (EH) and Morse Taper (MT) implants. Forty-four single implants were placed in the edentulous areas of 20 patients who met the inclusion and exclusion criteria. Immediately after implant placement (t1) and after osseointegration (four and six months for mandible and maxilla, respectively) (t2), insertion torque, resonance frequency, and peri-implant bone loss were measured using probing depths and digital periapical radiography. A significant difference was noted in the ISQ values between t1 and t2 in type III bone for EH and MT implants. No significant difference in bone loss values was observed when comparing bone types for EH or MT in all evaluated sites. Based on marginal bone loss assessed using radiography, there was no significant difference between the MT and EH groups. A positive correlation between torque and ISQ t1 value was observed for MT (correlation: 0.439; p = 0.041) and EH (correlation: 0.461; p = 0.031) implants. For EH and MT implants, the greater the insertion torque, the greater was the ISQ value (moderately positive correlation). A weak negative correlation was found between bone type and ISQ t1 for MT implants. Contrarily, no correlation was observed between bone type and ISQ t1 for EH implants. In all cases, bone loss around the implants was clinically normal.

## Introduction

Absence of one or more permanent teeth due to trauma or tooth agenesis is common among children and adolescents.^1^ Moreover, tooth loss is also common in adults, especially those aged > 50 years.^2^ This type of stomatognathic system impairment can lead to aesthetic and functional limitations (*e.g.*, eating and speech), and also impair psychological and social acceptance.^1,3^Dental implants have gained popularity as a satisfactory rehabilitation option for patients with missing teeth due to their ability to preserve the adjacent tooth and bone structures.^4,5^

Implant osseointegration has been defined as direct structural and functional connections between the bone and implant surface.^6,7^ Primary and secondary implant stabilities are fundamental for attaining successful osseointegration.^3,8^ The former is obtained immediately after implant placement, and its function is to prevent micromovements (above 150 μm) of the implant. Excessive micromotion reportedly results in encapsulation of the implant by fibrous tissue and consequently osseointegration failure.^3,8^ This type of stability is an important marker for determining the success or failure of implant osseointegration and verification of loading possibilities (late, early, or immediate) after implant placement.^3^ In contrast, secondary stability is obtained after bone growth and maturation over the implant, that is, after the osseointegration period.^3,7,8^ Notably, primary stability depends on several factors, including the amount of bone tissue in immediate contact with the implant surface, bone tissue quality around the implant,^8^implant design (length, diameter, shape, platform, and thread form/pitch), implant surface modification,^8,9^ and surgical technique.^8^

The most efficient, indicated, and used methods to evaluate primary stability of implants are resonance frequency analysis (RFA) and insertion torque.^3^ RFA was developed in the 1990s by Meredith et al., and it can be used to measure the primary and secondary stability of the implant, based on “implant vibration”.^3,6,10^RFA is evaluated using a device known as the Osstell mentor.^3,8^ To use this device, a component called “Smartpeg” is screwed onto the implant. The “Smartpeg” is electromagnetically excited at various frequencies (5–15 kHz) using a portable Osstell mentor probe.^3,8^ The resonance frequency (“vibration”) of the “Smartpeg” is then quantified in a unit of measurement called the “implant stability quotient” (ISQ), which ranges between 0 (minimum stability) and 100 (maximum stability).^3,8^Insertion torque was developed by Johansson and Strid,^12^ and improved by Friberg et al.^11^ in the 1990s.I It can only be used to evaluate the primary stability of the implant.^3,6,11,12^ It is measured in Ncm and is obtained with a torque meter at the end of implant placement in the bone.^3^ This method evaluates the frictional resistance encountered by the implant as it moves forward apically through a rotary movement in its axis.^3^

Once the implant is placed in the bone, bone remodeling initiates and is associated with marginal bone resorption around the implant.^13^ Marginal bone resorption can also occur due to numerous factors, including surgical trauma, biological width formation, abutment micromovement, plaque accumulation, inflammation in the microgap area, and occlusal trauma.^13^ Radiographic methods are widely used to assess marginal bone loss and osseointegration.^14^

Currently, the most commonly used implant platforms are Morse Taper (MT) and external hexagon (EH),^15^ both of which possess individual characteristics. For instance, the prosthetic connection mode is internal for the former and external for the latter.^15^ The idea behind MT is to have “a cone within a cone” that can generate mechanical locking, and in the field of dentistry, this is still coupled to a screw retention system.^15^ In contrast, EH platforms use a 0.7 mm-high hexagon that fits the abutment preventing its rotation, and the locking between these parts is screwed.^15^ Other differences between these types of implants include: a) distance between the alveolar bone and union between implant and abutment, which is present in MT implant but not in EH^15^and b) location of the implant platform after its placement in the bone, which is 1–2 mm apical to the bone crest for MT and at the level of the cortical bone for EH implants.^5^

Souza et al. reported contradictory findings regarding the correlation between insertion torque and RFA values.^3^ Thus, based on the information available at present, a PubMed search using the keywords “torque,” “implant stability quotient,” and “correlation” was performed to check for clinical articles comparing MT and EH implants in terms of correlation between insertion torque and ISQ. However, no studies have been published on this topic.

Therefore, the main objective of this study was to analyze the influence of insertion torque, recipient bone type, and peri-implant bone loss on the ISQ values of EH and MT implant connections. The study also aimed to evaluate the survival of the implants installed (EH and MT) as a secondary outcome.

## Methodology

### Ethical considerations

This study was approved by the Human Research Ethics Committee of Universidade Paulista (UNIP) through the “Plataforma Brazil” (https://plataformabrasil.saude.gov.br/login.jsf - Certificate of Presentation of Ethical Appreciation No. 91184918.6.0000.5512), and was carried out in accordance with the tenets of Declaration of Helsinki. All participants provided informed consent before their inclusion in the study. This study was registered on the REBEC platform (Brazilian Clinical Trials Registry - RBR-2qt66fq / Universal Trial Number - U1111-1285-0182).

### Study design

This study was a randomized controlled clinical trial. The volunteers were randomly allocated in a 1:1 ratio to either of the two interventional groups: EH or MT. One of the authors (D.M.S.), who was not involved in the selection process, generated the pseudorandom allocation sequence of volunteers’ ID using an available online tool (https://www.randomizer.org). The clinical trial was blinded, that is, volunteers, operators who performed the measurements, technicians who evaluated the radiographs, and outcome assessors were unaware of the allocation of the intervention. The present study complied with the CONSORT guidelines.

### Study population

Individuals who visited the Postgraduate Clinic of the Araçatuba Dental School in São Paulo State University (UNESP) between November 2017 and July 2018 formed our study population. Participants were included in this study based on the inclusion and exclusion criteria. The study included patients with good oral health who required rehabilitation with single dental implants (11.5 × 4 mm) in edentulous spaces (> four months) between two teeth with a maximum distance of 1.5–2 mm between tooth and implant. The bone types included for evaluation were types II and III according to the Lekholm and Zarb classification.^3^ Individuals with history of periodontal disease, those with periapical lesion in the tooth adjacent to the edentulous area, those who required bone grafting and/or use of a barrier membrane for implant placement, those with metabolic bone diseases, and those who received anticoagulants or bisphosphonate for a prolonged period were not included in this study. Additionally, immunocompromised individuals and those who smoked were also excluded from the study.

Sample size was determined based on a pilot study involving six patients. The test was based on mean RFA outcomes of 86.2 and 76.6 in the EH and MT groups, respectively; a standard deviation of the outcome of 6.3 with 95% confidence level; 80% power; and medium effect size. The calculation results showed that seven individuals per group were required for the study.

**Figure 1:**
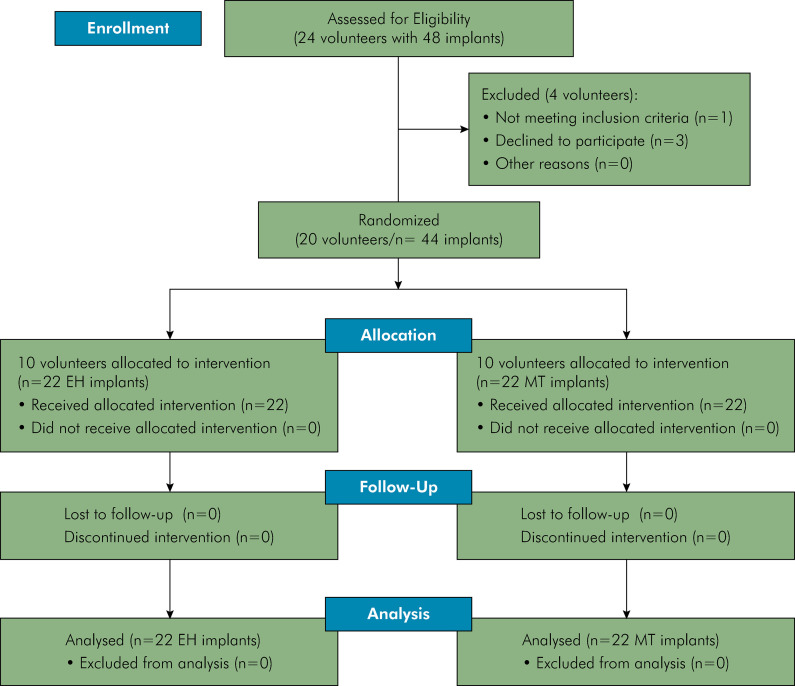
The CONSORT flow diagram.

Based on the inclusion and exclusion criteria, the study included a total of 20 volunteers with 44 edentulous areas. Forty four implants were placed, with 22 each of EH and MT types (Figure 1). Volunteers were divided into two groups according to the implant type: EH (10 volunteers) and MT (10 volunteers) groups.

### Surgical protocol

Periapical radiographs (Spectro 70X Seletronic, Dabi AtlanteS/A Indústrias Médico Odontológica Ltda, Ribeirão Preto, Brazil), panoramic radiographs (digital Pax-400, Vatech do Brasil, Vatech Co. Ltda, São Paulo, Brazil), and cone-beam computed tomography (CBCT - Soredex 3DX, Soredex, Helsinki, Finland) of the edentulous areas of the patients were performed and evaluated by an experienced surgeon (M.C.G.).^16^ CBCT images were acquired to verify the bone height and thickness of the edentulous area and plan the implant position. CBCT scans were performed with an adjusted field of view (FOV) of 10 × 8 cm, voxel size of 0.15 mm, acquisition time of 15 s, 90 kVp, and 10 mA – High-Definition Protocol. Furthermore, laboratory investigations (complete blood count, coagulogram, urea, creatine, and fasting glucose) were requested from the patients before the implant placement surgery. Patients were referred to a physician to control systemic changes, if any.

After performing implant position planning, surgical guides were made similar to those in the study by Becker and Kaiser.^17^Before implant placement, the vital signs (blood pressure and heart rate) of the patients were checked. If they were within the normal limits, drug prophylaxis (100 mg of nimesulide and 1 g of amoxicillin or azithromycin, depending on the patient’s allergenicity to penicillin) was initiated 1 h before surgery. Subsequently, patients were instructed to rinse their mouth with 0.12% chlorhexidine (Periogard, Colgate, Brazil) for 1 min.^18^ Subsequently, facial antisepsis was performed using 1% iodopovidone (Riohex 4%, Rioquímica Indústria Farmacêutica, São José do Rio Preto, Brazil), placement of sterile operative field, and anesthesia (Articaine 100, Articaine HCL 4% with epinephrine 1:100000, DFL, Rio de Janeiro, Brazil).^18^ All surgeries were performed by the same operator (M.C.G.) and all implants were placed according to the manufacturer’s recommendations using the same surgical technique. EH implants were placed at the bone crest level, whereas MT implants were placed 1 mm below the bone crest.^5,19^After performing the evaluations (torque and ISQ values), implants were covered with cover screws for the duration required for osseointegration.

Type of bone for implant placement was classified by the operator (M.C.G.) according to the Lekholm and Zarb classification.^3,20^This classification is based on the analysis of bone quantity and quality in the field of dental implants using preoperative radiographs (panoramic and periapical) and exploratory drilling during implant site preparation. The analyzed bone quality comprises four groups depending on the amount of compact and spongy bone present.^3,20^

Postoperative medication (one capsule of 500 mg amoxicillin every 8 h for 7 days or one tablet of 500 mg azithromycin every 24 h for 5 days and one tablet of 100 mg nimesulide every 12 h for 3 days) was prescribed to the patients.^18^

A total of 44 implants, including 22 EH (Biofit, DSP Biomedical, Campo Largo, Brazil) and 22 MT (Biofit Indexed, DSP Biomedical, Brazil) implants were used. All implants were made of pure titanium (grade IV), and had the same surface design, retention grooves, dimension of 11.5 × 4 mm, and surface treatments. According to the manufacturer, surface treatment was performed using acid etching, which characterizes the subtraction process, thereby forming microvalleys.

### Measuring procedure

#### Insertion torque and ISQ

Implant stability was measured using insertion torque and RFA. A calibrated manual torque meter (DSP Biomedical, Campo Largo, Brazil) was used to measure insertion torque^3^. Maximum torque value was obtained upon final positioning of the implant within the bone, and its measurement unit was Ncm.^3,21^

RFA of each implant was assessed using an Osstell device (Osstell Mentor, Göteborg, Sweden) immediately after implant placement (t1) (primary stability) and after the osseointegration period (four months for mandible and six months for maxilla) (t2) (secondary stability).^22^ Implants inserted in the bone were coupled to a SmartPegTM transducer, which was specific for each implant type.^3,23^ The Osstell measuring rod was brought close (approximately 1 mm) to the SmartPegTM and stimulated using magnetic pulse emission. This resulted in the transducer to resonate at specific frequencies on the basis of the stability level of the implant.^3,23^ Thereafter, the device emitted a beep and displayed the RFA value.^3^ Four readings (mesial, distal, buccal, and lingual/palatal) were noted, with the Osstell measuring rod positioned at four different points on the SmartPegTM attached to the implant for analysis.^3^ Subsequently, an average of four implant ISQ values was obtained.^3^

#### Peri-implant bone loss

**Figure 2:**
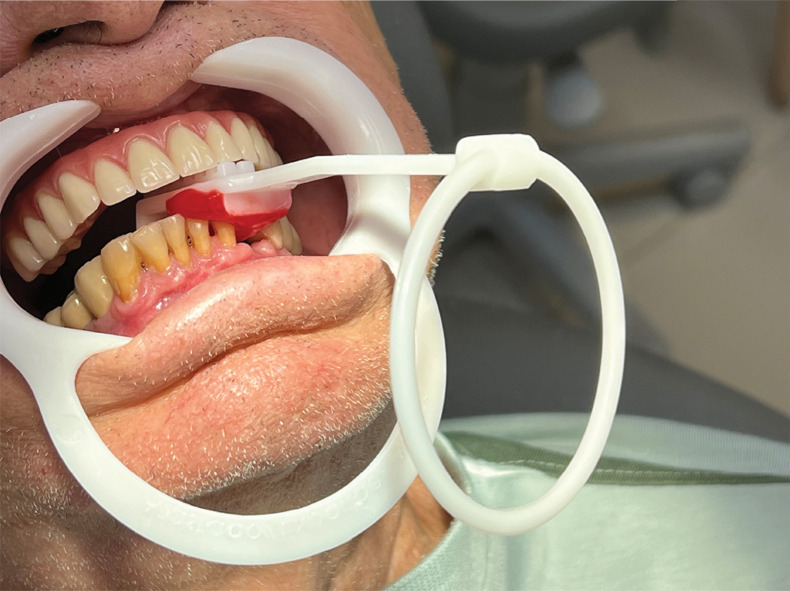
Device for standardizing radiographs.

**Figure 3:**
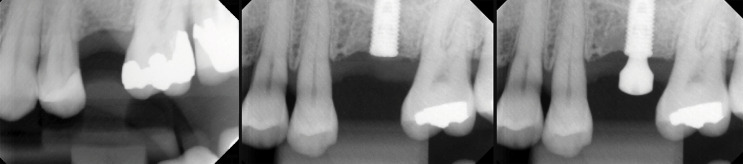
Standardized radiographs for measuring crestal bone loss. Left image - initial (edentulous area without implant); center image - after implant placement; and right image - after osseointegration of the implant.

Marginal bone loss was assessed radiographically and through probing immediately after implant placement (t1) and after osseointegration (t2). An oral and maxillofacial radiologist (with 27 years of practical experience – L.M.P.S.) analyzed the periapical radiographs that were taken with the same exposure time (0.2 s) and periapical radiograph device operating at 70 kVp (8mA) (Spectro 70X Seletronic, Dabi AtlanteS/A Indústrias Médico Odontológica Ltda, Ribeirão Preto, Brazil). The complementary metal oxide semiconductor (CMOS) digital radiography system used was a Microimagem/EVO sensor (Acteon/Microimagem, Indaiatuba, Brazil), sensor size 2, with an external area of 31 × 41 mm, an active area of 26 × 34 mm, and a theoretical resolution of 20 pl/mm (26line pairs). To standardize the radiographic images, diagnostic casts were made from alginate impressions (Ezact Kromm, Vigodent, Bonsucesso, Rio de Janeiro, Brazil) using quadrant impression trays to make chemically activated acrylic resin guides (Pattern Bright, Kota, Cotia, Brazil). A specific guide was created for each edentulous area of each patient. This guide was fitted to the teeth adjacent to the edentulous area, and a radiographic positioner was fitted to this guide (Indusbello, Londrina, Brazil) (Figures 2 and 3).

The focus/film distance was standardized to 30 cm. The fitting of the acrylic resin device to the positioner allowed for a standardized reproduction of the radiographs. The radiographs were analyzed by a specialist in a controlled lighting environment using an HP All-in-One Omni 200 PC monitor (Hewlett-Packard, Palo Alto/CA, USA) with a display resolution of 1920 × 1080 pixels, 21.5 inches diagonal, and 300× magnification. Bone loss was evaluated using digital image processing to facilitate visualization of the anatomical structures of interest. Image processing and analysis were performed using ImageJ software (Freeware, http://www.rsbweb.nih.gov) that is based on Java programming language. Peri-implant linear bone loss was measured by comparing the initial (t1, immediately after implant placement) and final (t2, after osseointegration) radiographic images. The set scale tool was used as a horizontal reference to calibrate the software, and the straight tool was used to adopt the distance between two implant threads as a vertical reference.^24^ The measurement tool was used to obtain the values of the respective measurements, which were saved in TIFF format in different files, corresponding to the radiographs at t1 and t2.

Bone loss, evaluated by probing depth, was measured using a precisely graduated periodontal probe (Hu-Friedy Mfg. Co., LLC., Frankfurt, Germany). The assessment was performed at two time points (t1, immediately after implant placement and t2, after osseointegration). Probing depth was measured at buccal, palatal/lingual, mesial, and distal implant sites. Negative or positive values were assigned, depending on whether the implant platform was above (positive) or below (negative) the bone crest.

#### Implant survival criteria

Implant survival was assessed based on the absence of painful symptoms, mobility, peri-implant radiographic radiolucency, and progressive marginal bone loss.^3,25,26^ Moreover, RFA value was also used to verify implant stability over time.^3^

## Statistical analysis

Statistical analyses were performed using IBM SPSS 24.0 software (Statistical Package for the Social Science, SPSS Inc., Chicago, USA). Analysis of normality, which was observed in the ISQ data, was performed using the Shapiro-Wilk test. Based on the type of implant placed in different bone types and torque used in different implant types, ISQ values over time were evaluated via repeated measures three-way analysis of variance (ANOVA), followed by Tukey’s test. Two-way ANOVA and Tukey’s test were used to compare the insertion torque for each implant type between the bone types. For each implant type, torque data were correlated with the initial and final ISQ measurements using Pearson’s correlation. Implant site bone type and torque classification data were separately correlated with initial and final torque and ISQ measurements using Kendall’s correlation for EH and MT implants. Based on the radiographic analysis of marginal bone loss, data were subjected to Student’s t-test. Analysis of bone loss at implant margin in millimeters (millimeter probe method) at different sites (mesial, distal, buccal, and lingual/palatine) for different implant placements based on the bone type was performed using Kruskal-Wallis test. Additionally, ISQ data at initial time point (t1) were analyzed based on implant type and amount of peri-implant bone loss (no bone loss and bone loss up to 1, 2, or 3 mm) at different sites (mesial, distal, buccal, and lingual/palatal). All analyses were performed at 5% significance level.

## Results

A total of 20 volunteers (10 females and 10 males) with 44 edentulous areas were included in this study. Twenty-two MT (9 in type III and 13 in type II bone) and 22 EH (9 in type III and 13 in type II bone) implants were placed. After osseointegration of the maxilla (six months) and mandible (four months), the success/survival rate of the implants was 100%. The mean age of the volunteers rehabilitated with EH and MT implants was 45.54 years and 47.38 years, respectively (Table 1).

**Table 1:** Baseline demographic and clinical characteristics of the study groups.

Variable	External hexagon (EH)	Morse taper (MT)
Gender (male/female)	3/7	5/5
Mean age (years; SD)	45.54 (8.6)	47.38 (9.3)
Number of implants	22	22
Implant brand	DSP Biomedical	DSP Biomedical
Location (Maxilla/mandible)	6/16	7/15
Type Bone (II/III)	13/9	13/9
Success/Survival (%)	100	100

Three-way ANOVA (p < 0.05) applied to the ISQ values of the placed implants based on bone type showed a significant difference in the context of the following factors: “Implant” (p = 0.013), “Time” (P < 0.001), and “Time × Bone” (p < 0.001). For type III bone, there was a significant increase in the ISQ value at t2 for both types of implants (Table 2). Comparison of ISQ t1 (bone type II) with ISQ t1 (bone type III) revealed that the ISQ t1 (bone type III) value was significantly lower (Table 2). Furthermore, comparing EH with MT showed that significant differences existed for only type III bone (Tukey; p < 0.05; Table 2): “ISQ t1 - EH” × “ISQ t1 - MT” (a higher ISQ value for the EH); and “ISQ t2 - EH” × “ISQ t2 - MT” (a higher ISQ value for the EH).

Three-way ANOVA (p < 0.05) applied to the ISQ values of the placed implants based on insertion torque showed a significant difference in the context of the following variation factors: “Time” (p < 0.001) and “Time × Torque” (p < 0.001). ISQ values at t2 were significantly higher than those at t1 for both implants when the torque range was 30–44 Ncm (Table 3). Similar situation was observed for torque condition of ≥ 45 Ncm only for MT implants. Furthermore, comparing EH with MT revealed that only the following significant differences were observed (Tukey; p < 0.05; Table 3): Torque ≥ 45 Ncm -”ISQ t1 - EH” × “ ISQ t1 - MT” (a higher ISQ value for the EH) and “ISQ t2 - EH” × “ ISQ t2 - MT” (a higher ISQ value for the EH).

**Table 3:** Mean RFA values for different implants placed based on the insertion torque and analysis time.

Torque classification	Number of total implants (EH/MT)	RFA			

		External hexagon (EH)		Morse taper (MT)	

		ISQ t1	ISQ t2	ISQ t1	ISQ t2
30–44	10 (7/3)	72.18 ± 9.73^Aa^	81.75 ± 9.22^Ba^	63.92 ± 14.08^Aa^	78.33 ± 5.27^Ba^
≥ 45	34 (15/19)	79.22 ± 7.33^Aa^	81.77 ± 4.78^Aa^	72.29 ± 11.08^Aa^	75.76 ± 8.50^Ba^

Same lower-case letters in the column and upper-case letters in the row (for each implant type separately) indicate that the means do not differ significantly (p < 0.05, Tukey test). RFA: resonance frequency analysis.

Insertion torque values of the placed implants based on bone type were significantly different after a two-way ANOVA (p < 0.05) for the following variation factors: “Bone type” (p < 0.001) and “Implant” (p = 0.03). For MT and EH implants, significantly higher torque values were observed for type II bone than type III bone (p < 0.05) (Table 4).

**Table 4:** Mean insertion torque values (Ncm) for different implants placed based on the bone type.

Bone type	Number of implants	Insertion torque	

		External hexagon (EH)	Morse taper (MT)
Type II	26	53.08 ± 5.60^Aa^	54.62 ± 1.39^Aa^
Type III	18	37.22 ± 6.18^Ab^	42.78 ± 6.67^Bb^

Different lowercase letters in the column represent a statistically significant difference. Upper case letters in the row represent a statistically significant difference (p < 0.05, Tukey test).

For EH implants, correlation of insertion torque data with ISQ values at t1 (correlation: 0.461; p = 0.031), bone type (correlation: -0.777; p < 0.001), and torque classification (correlation: 0.805; p < 0.001) was noted. However, there was no correlation between insertion torque and ISQ t2 (p = 0.876). Furthermore, correlations were also observed between ISQ values at t1 and t2 (correlation: 0.738; p < 0.001); and torque classification and bone type (correlation: -0.623; P = 0.004). No correlations between ISQ values at t1 and bone type (p = 0.256), ISQ t1 and torque classification (p = 0.084), ISQ t2 and bone type (P = 0.316), and ISQ t2 and torque classification (p = 0.860) were noted.

MT implants exhibited correlation of insertion torque values with ISQ at t1 (correlation: 0.439; p = 0.041), bone type (correlation: -0.805; p < 0.001), and torque classification (correlation: 0.638; p = 0.002). However, there was no correlation between insertion torque and ISQ t2 (i = 0.757). Furthermore, correlations between ISQ values at t1 and at t2 (correlation: 0.728; p < 0.001), torque classification and bone type (correlation: -0.478; p = 0.029), and ISQ t1 and bone type measurements (correlation: -0.366; p = 0.045) were noted. No correlation was observed between ISQ t1 and torque classification (p = 0.232), ISQ t2 and bone type (P = 0.422), or ISQ t2 and torque classification (p = 0.962).

Bone loss evaluation using radiography showed no significant difference (Student’s t-test: p = 0.745) between EH (0.08 ± 0.07) and MT implants (0.08 ± 0.07). Based on the probing method, for EH or MT, there was no difference between type III and II bone in terms of bone loss, regardless of the bone site evaluated.

## Discussion

Drilling technique, friction coefficient during implant placement, homogeneity of the implantation site, and implant factors such as shape (tapered or cylindrical), diameter, surface micro-roughness, length, thread format, presence of retentive grooves, and surface modifications can influence RFA and insertion torque values.^27,28^ Thus, in the present study, all implant placement surgeries were performed by the same operator using the same surgical technique and at sites with similar characteristics to avoid bias. Furthermore, implants with same surface treatment, body/design, dimensions, and retentive characteristics (grooves) were standardized.

As shown in Table 2, for type III bone, a significant increase in ISQ value at t2 was observed for both types of implants. For type II bone, no difference between ISQ t1 and ISQ t2, regardless of implant type, was noted (Table 2). This can be attributed to the fact that type II bone has a larger cortical layer (which is denser than the medullary bone) and generates larger contact surface for the implant in comparison to type III bone.^3,20^This possibly contributed to the primary stability values being similar to the secondary stability values of both types of implants for type II bone (P > 0.05). Therefore, clinically, for type II bone, dentists should not expect any significant increase in implant stability after osseointegration; however, for type III bone, it is possible to expect a significant increase in implant stability after osseointegration.

When comparing the mean values of ISQ t1 (primary stability) for MT or EH implants (Table 2), it is possible to verify that the value of ISQ t1 for MT in bone type III was significantly lower compared to the value of ISQ t1 for MT in bone type II. In contrast, for EH, there was no significant difference in the ISQ t1 values (type III bone vs. type II bone; p > 0.05). This suggests that the vibration stability (determined using RFA) of an implant may be influenced by the type of surrounding bone and the depth of the implant within the bone. It is likely that the MT implant, which was placed 1 mm below the bone crest, lost part of the benefit of being stabilized by the cortical part of type III bone. However, this did not occur in the case of type II bone due to the greater thickness of its cortical part. In contrast, EH implant placed at the level of the bone crest was favored by being stabilized throughout the cortical part of the type III bone. Furthermore, when comparing ISQ t1 (EH) with ISQ t1 (MT), there was a significant difference only for type III bone, in which EH presented a significantly higher ISQ value, corroborating this situation (Table 2). These results showed that type III bone favored the stability of EH implant more than the stability of MT implant as per the RFA method. It is worth mentioning that the RFA method evaluates the vibration of the implant placed in the bone, therefore, the amount of cortical bone around the implant proved to be very important for greater primary stability.

In the context of secondary stability (Table 2), when comparing ISQ t2 (EH) with ISQ t2 (MT), significant difference was observed only for type III bone, in which EH presented a significantly higher ISQ value. This suggests that after osseointegration, bone type may also influence the vibration stability of the implant depending on its depth (EH or MT).

Table 3 shows the RFA values based on torque classification and analysis time, regardless of the bone type. Thus, it is possible to verify that when the torque was < 45 Ncm,^3^ there was a significant increase in ISQ t2 value for both implants (ISQ t2 vs. ISQ t1). In contrast, when the torque was ≥ 45 Ncm,^3^ only MT group showed a significant increase in ISQ value at t2 (Table 3). Clinically, dentists can expect that when the torque is less than 45 Ncm, the ISQ value will increase significantly after the implant osseointegration period. However, when the torque is ≥ 45 Ncm, the ISQ value may or may not increase significantly after this period.

Evaluation using insertion torque method revealed significantly higher torque values in type II bone than in type III bone, regardless of the type of implant used (Table 4). This could also be explained by the greater amount of cortical bone in type II bone than in type III bone, which generated greater primary stability in both types of implants.^3,20^

MT in type II bone showed significantly greater torque than MT in type III bone (Table 4). Furthermore, EH in type II bone showed a significantly greater torque than EH in type III bone. Thus, using the torque method, type III bone did not favor the stability of EH implant, unlike what occurred with the RFA method. This may have occurred because RFA (implant vibration) and torquemeter (frictional resistance) evaluate different aspects of primary stability.

This study showed a positive correlation between insertion torque and ISQ t1 value for MT (correlation: 0.439; p = 0.041) and EH (correlation: 0.461; p = 0.031) implants. Thus, the higher the ISQ t1 value, the higher is the insertion torque value, and vice versa. These correlation coefficient values were classified as moderate.^29^ Thus, both types of implants showed a moderate positive correlation between torque and ISQ t1.^29^

For both implant types, strong negative correlations^29^ between insertion torque and bone type (EH: -0.777, p < 0.001; MT: -0.805, p < 0.001) were noted. Thus, the higher the insertion torque (EH or MT), the lower will be the bone type classification (based only on bone types II and III), and vice versa. The bone classification system given by Lekholm and Zarb^20^ can explain this result as lower the number in this classification, the greater is the amount of cortical bone present, and consequently, the greater the chance of achieving higher primary stability of the implant.^3,20^ Souza et al.^3^ also found a comparable situation based on bone types I and III upon evaluating the implants placed in the alveolus immediately after tooth extraction.^3^

MT implant showed a weak negative correlation^29^ between bone type and ISQ t1 value (correlation: -0.366; p = 0.045). No significant correlation was observed between bone type and ISQ t1 value for EH implant (p = 0.256). These different results may be related to the differences between the internal designs (and platforms) of these implants, as well as their different depths in the bone.^5,7,15,19^ Despite this, these two results show that the RFA method is inadequate for clinical assessment of bone quality, unlike the insertion torque method (different characteristics of primary stability assessment might explain this situation: RFA, vibration of the implant and insertion torque, and frictional resistance during implant threading).^3^

Although the present study did not evaluate implants after prosthetic rehabilitation (implants under the influence of occlusal load), it was possible to use the mean ISQ t1 and torque values to discuss the indications for implant loading. Insertion torque and ISQ t1 values based on single crowns could be interpreted as follows: a) insertion torque values < 30 Ncm represent low implant stability (indication for late implant loading), b) insertion torque values ranging between 30–44 Ncm represent medium implant stability (indication for late implant loading), c) insertion torque values ≥ 45 Ncm represent high implant stability (indication for immediate, early or late implant loading); a) ISQ values < 60 represent low implant stability (indication for late implant loading), b) ISQ values ranging between 60–64 represent medium-low implant stability (indication for late implant loading), c) ISQ values between 65–69 represent medium-high implant stability (indication for early or late implant loading); and d) ISQ values ≥ 70 represent high implant stability (indication for immediate, early, or late implant loading).^3^ The aforementioned findings highlight that insertion torque and ISQ t1 values might indicate different implant loading possibilities. Thus, the following situations could be observed: 1) with the exception of one ISQ t1 value (64.69) for MT implant in type III bone (Table 2), which indicated only delayed loading, all other mean ISQ t1 values would allow immediate, early, or delayed loading of the implants (Table 2) and 2) insertion torque values > 45 Ncm were observed only in type II bone for both implant types, indicating the three treatment possibilities (immediate, early, or delayed loading), unlike the torque values for both types of implants in type III bone, which were < 45 Ncm, indicating only late loading (Tables 2 and 4). Thus, on more occasions, RFA method indicated more treatment possibilities than insertion torque. An analogous situation was also observed in a study by Souza et al.,^3^ who recommended adopting early or immediate loading only when both methods indicated this possibility. It is noteworthy that, in this study, all mean ISQ t2 values (> 70 ISQ) demonstrated high secondary stability of the implants evaluated (Table 2).

Marginal bone loss of up to 2 mm around the implant after the first year of its placement is considered clinically normal.^7,24,30^ In the present study, bone loss occurring around EH and MT implants was evaluated using two different methods (radiographic and probing). In this study, although the evaluation period was less than 1 year, the average bone loss values according to the radiographic method did not exceed 0.08 mm in the EH and MT groups. Therefore, these values were considered clinically normal. It is worth noting that, based on marginal bone loss assessed by the radiographic method, there was no significant difference between the MT and EH groups.

During the osseointegration period, bone loss in height may occur around the implant,^7,24,30^ and depending on the depth of implant placement, the external surface (implant body) may or may not get exposed. In this study, MT showed an advantage over EH in this situation because its platform was located 1 mm below the bone crest after its placement (Table 5). Thus, Table 5 shows that most EH implants had their external surfaces exposed after osseointegration, unlike MT implants, which mostly did not have their external surfaces exposed after this period.

**Table 5:** Mean of bone loss (mm) at different sites (mesial, distal, buccal, and lingual/palatal) for the different placed implants based on the bone type.

Bone type	Bone loss (probing in mm) per site											

	Mesial			Distal			Vestibular			Lingual/palatal		

	EH	MT	p-value	EH	MT	p-value	EH	MT	p-value	EH	MT	p-value
Type II	0.5 ± 0.5	-0.1 ± 0,7	0.023*	-0.1 ± 0.6	0.1 ± 0.9	0.730	0.2 ± 0.6	0.1 ± 0.6	0.533	0.1 ± 0.3	-0.2 ± 0.5	0.179
Type III	0.3 ± 0.7	-0.7 ± 0,5	0.007*	0.0 ± 0.9	0.0 ± 1.3	0.675	0.4 ± 0.5	-0.3 ± 0.5	0.010*	0.2 ± 0.4	-0.3 ± 0.5	0.029*
p-value	0.734	0.076		0.827	0.520		0.417	0.127		0.340	0.461	

*Kruskal-Wallis test was performed for comparison between EH and MT implants (p < 0.05). EH: External hexagon; MT: Morse taper.

Based on the probing method, bone loss values were considered clinically normal (Table 5).^7,24,30^ Furthermore, for EH or MT at all sites evaluated (mesial, distal, buccal, and lingual/palatal), when comparing type III bone with type II bone, no significant difference was noted in the bone loss values. Thus, the evaluated bone types did not affect the amount of bone lost.

Limitations of the present study include lack of evaluation of bone types I and IV, comparison of implants with different surface treatments, and use of other types of implant platforms. Furthermore, the measurements were performed without an occlusal load. Therefore, future studies are necessary to evaluate these topics in addition to evaluating the maxilla and mandible, as well as the male and female sexes, separately.

## Conclusion

For EH and MT implants, the greater the insertion torque, the greater the ISQ value (moderately positive correlation). For MT implant, there was a weak negative correlation between bone type and ISQ t1 value. No correlation was observed between bone type and ISQ t1 value for EH implant. Bone loss around the implants was normal in all cases (evaluated using probing and radiographic methods).

**Table 2:** Mean RFA values for different implants placed based on the bone type, insertion torque, and analysis time.

Bone type	Number of implants	Insertion torque	RFA			

			External hexagon (EH)		Morse taper (MT)	

			ISQ t1	ISQ t2	ISQ t1	ISQ t2
Type II	26	53.85 ± 4.08	78.90 ± 6.62^Aa^	80.73 ± 4.72^Aa^	75.61 ± 8.17^Aa^	77.38 ± 7.41^Aa^
Type III	18	40.00 ± 6.86	74.19 ± 10.68^Aa^	83.25 ± 8.13^Ba^	64.69 ± 13.00^Ab^	74.28 ± 9.12^Ba^

Same lower-case letters in the column and upper-case letters in the row (for each implant type separately) indicate that the means do not differ significantly (p < 0.05, Tukey test). RFA: resonance frequency analysis.
